# Highland Barley Alleviates High-Fat Diet-Induced Obesity and Liver Injury Through the IRS2/PI3K/AKT Signaling Pathway in Rats

**DOI:** 10.3390/nu16203518

**Published:** 2024-10-17

**Authors:** Xiaodong Shi, Wei Song, Boyue Jiang, Jie Ma, Wanyang Li, Mingyao Sun, Hongyuan Cui, Wei Chen

**Affiliations:** 1 Beijing Key Laboratory of the Innovative Development of Functional Staple and the Nutritional Intervention for Chronic Disease, Department of Clinical Nutrition, Department of Health Medicine, Peking Union Medical College Hospital, Peking Union Medical College, Chinese Academy of Medical Sciences, Beijing 100730, China; zs201910@student.pumc.edu.cn (X.S.); 202462000650@email.sdu.edu.cn (W.L.); pumc_2022sunmingyao@student.pumc.edu.cn (M.S.); 2 Center for Biomarker Discovery and Validation, Institute of Clinical Medicine, Peking Union Medical College Hospital, Peking Union Medical College, Chinese Academy of Medical Sciences, Beijing 100730, China; songwei51@pumch.cn; 3 Department of Hepato-Bilio-Pancreatic Surgery, Department of General Surgery, Beijing Hospital, National Center of Gerontology, Institute of Geriatric Medicine, Peking Union Medical College, Chinese Academy of Medical Sciences, Beijing 100730, China; pumcjby@student.pumc.edu.cn; 4 Department of Hepatopancreatobiliary Surgery, The Affiliated Hospital of Qinghai University, Xining 810001, China; 1996900012@qhu.edu.cn

**Keywords:** highland barley, obesity, HFD, liver injury

## Abstract

**Objectives:** Highland barley (HB) consumption offers numerous health benefits; however, its impact on glycolipid metabolism abnormalities induced by a high-fat diet remains unclear. Consequently, this study aimed to investigate the therapeutic effects and underlying molecular mechanisms of HB in the context of obesity; **Methods:** Rats were fed either a high-fat diet (HFD) to induce obesity or a standard diet (SD) for six weeks. The rats in the HFD group were randomly assigned into five groups: HFD+HFD, HFD+SD, and low (30%), medium (45%), and high (60%) doses of the HB diet for an additional ten weeks. Analyses of serum lipid profiles, liver histology, transcriptomes, and untargeted metabolomes were conducted; **Results:** HB intake resulted in decreased weight gain, reduced feed intake, lower serum triglyceride and cholesterol levels, and diminished hepatic lipid accumulation. It also improved insulin and fasting blood glucose levels, and antioxidant capacity in the HFD-fed rats. Transcriptome analysis revealed that HB supplementation significantly suppressed the HFD-induced increase in the expression of Angptl8, Apof, CYP7A1, GDF15, Marveld1, and Nr0b2. Furthermore, HB supplementation reversed the HFD-induced decrease in Pex11a expression. Untargeted metabolome analysis indicated that HB primarily influenced the pentose phosphate pathway, the Warburg effect, and tryptophan metabolism. Additionally, integrated transcriptome and metabolome analyses demonstrated that the treatments affected the expression of genes associated with glycolipid metabolism, specifically ABCG8, CYP2C12, CYP2C24, CYP7A1, and IRS2. Western blotting confirmed that HB supplementation impacted the IRS2/PI3K/AKT signaling pathway; **Conclusions:** HB alleviates HFD-induced obesity and liver injury in an obese rat model possibly through the IRS2/PI3K/Akt signaling pathway.

## 1. Introduction

Obesity constitutes a significant public health issue, imposing an escalating social and health burden globally. According to the World Obesity Atlas, 2023, the number of individuals with obesity (BMI ≥ 25 kg/m^2^) worldwide is expected to exceed 4 billion by 2035, which will be more than half of the global population. Obesity can lead to several diseases that cause huge economic burdens on individuals, families, and the society. For example, the economic burden of overweight and obesity in 2020 was USD 1.96 trillion, and is expected to exceed USD 4 trillion by 2035. Specifically, the economic implications include the healthcare expenditure of treating obesity and its resulting complications. Furthermore, high BMI contributes to increased absenteeism, presenteeism, as well as premature retirement or mortality [1]. Obesity, characterized by an abnormal or excessive buildup of fat, serves as a risk factor for several chronic diseases, including type 2 diabetes mellitus (T2DM), metabolic syndrome, cardiovascular diseases and cancers [2].

Highland barley (HB) has a long cultivation history in China, and is cultivated in various provinces in the northwest and southwest, even under cool plateau climates. HB has high protein, fiber, and vitamin contents, and contains several phytochemicals, such as β-glucan, polyphenols, flavonoids, phenolic, and minerals [3]. β-Glucan can inhibit the synthesis of cholesterol in the liver, enhance low-density lipoprotein clearance, and reduce cholesterol levels. Additionally, β-glucan absorbs water and expands in the intestinal tract, which increases the digestive tract viscosity, affects the absorption of fat, cholesterol, and other lipid substances, delays gastric emptying, reduces glucose absorption, and alleviates pancreatic islets β cell burden [4]. Polyphenols possess the capacity to inhibit the activities of α-amylase and α-glucosidase, stimulate insulin secretion by influencing glucose transporter actions, counteract oxidative damage and inflammation induced by hyperglycemia, and enhance blood glucose regulation [5].

The liver is crucial for maintaining systemic homeostasis and serves as a primary site for glycolipid metabolism. Obesity is often associated with abnormal gene expression related to carbohydrate, lipid, and protein metabolism, particularly those involved in pathways linked to insulin resistance, as well as fatty acid and amino acid synthesis. Insulin resistance, which frequently occurs in obesity, promotes fat accumulation in liver cells by enhancing fat breakdown and elevating insulin levels [6]. Insulin receptor substrates (IRSs) play a crucial role in insulin signaling and are essential for the progression of insulin resistance. There are several members in the IRS family, such as IRS1, IRS2, IRS3, and others. Among these, IRS2 is particularly important for liver homeostasis by mediating insulin synthesis and metabolism through the phosphatidylinositol 3-kinase (PI3K)-protein kinase B (AKT) signaling pathway. Additionally, it inhibits gluconeogenesis and cell apoptosis [7]. Furthermore, the IRS2/PI3K/AKT is a principal signaling pathway engaged in the regulation of glucose transport and metabolism [8,9,10,11,12].

Recent investigations into the positive impacts of HB on the glucose metabolism has achieved promising results; however, the molecular mechanisms remain unclear. Establishing an animal model with characteristics similar to those of human obesity is particularly important for exploring the pathological mechanisms and conducting basic research on interventions. However, the complex mechanisms underlying the glycolipid metabolism in hepatocytes remain not fully understood. A comprehensive understanding of how natural anti-obesity foods influence hepatic metabolism in both healthy and diseased states would aid in the creation of novel therapeutic strategies for obesity and associated chronic illnesses.

Consequently, the objective of this study was to explore the anti-obesity effects and potential mechanisms of HB in a rat model of obesity. Notably, this study may provide a theoretical foundation for the advancement of HB as a functional food.

## 2. Materials and Methods

### 2.1. Materials and Reagents

The HB variety used in this study was “Kunlun 14” (Academy of Agriculture and Forestry Science, Qinghai, China). The chemical/nutritional composition of the grain is as follows: carbohydrate, 58.50%; protein, 11.08%, fat, 3.00%; total dietary fiber content, 16.30 g/100 g; β-glucan, 4.90 ± 0.23%; total flavonoids, 0.05 ± 0.0025%; total polyphenols, 123.49 ± 5.39 mg/100 g; and pentosan, 13.76 ± 0.39 g/100 g.

### 2.2. Animals, Diets, and Experimental Design

**Figure 1:**
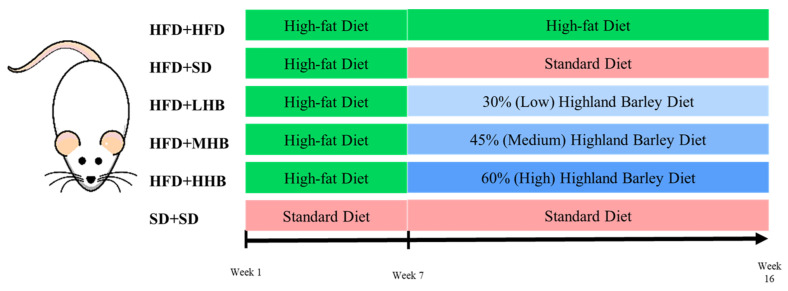
Animal study design. HFD+HFD group (maintained on D12492 diet throughout), HFD+SD group (administered D12492 diet for 6 weeks, subsequently transitioned to a standard diet), HFD+LHB group (administered D12492 diet for 6 weeks, then shifted to a 30% highland barley diet), HFD+MHB group (administered D12492 diet for 6 weeks, then switched to a 45% highland barley diet), HFD+HHB group (administered D12492 diet for 6 weeks, followed by a switch to a 60% highland barley diet), SD+SD group (consistently fed with a standard diet.).

Thirty-six male Sprague–Dawley rats were obtained from Shanghai JieSiJie Laboratory Animal Co., Ltd. (Shanghai, China; license # SCXK(Hu) 2023-0004). All rats were maintained in a controlled environment, featuring a constant temperature of 22 ± 2 °C and a 12 h light/dark cycle. After a 1-week acclimatization period, the rats were assigned to six groups (*n* = 6/group). Rats in the standard diet (SD+SD) group were fed a standard diet continuously. Rats in the remaining groups were fed a continuous high-fat diet (HFD) for a duration of 6 weeks, during which they were intraperitoneally injected with 40 mg/kg streptozotocin (STZ; Sigma-Aldrich, St. Louis, MO, USA) on the 5th day. Rats in the SD+SD group were simultaneously given an equivalent volume of saline. After the obesity model was successfully established, the rats were randomly divided into the following 5 groups: HFD+HFD (continued on HFD [D12492; 60% fat]), HFD+SD (continued on SD), and low (30%; HFD+LHB), medium (45%; HFD+MHB), and high (60%; HFD+HHB) HB supplementation groups (Figure 1). HFD and SD were provided by Fanbo Biotechnology Co., Ltd. (Nanjing, China), and the HB diets were processed by Huafukang Biotechnology Co., Ltd. (Beijing, China). The compositions of the experimental diets are detailed in Table 1. HB diets were formulated by substituting components of the standard diet with varying levels of HB as carbohydrate, protein, and fat source in order to maintain an approximately equivalent caloric value. The rats were fed these experimental diets for an additional 10 weeks. Body weight was recorded weekly and feed consumption was monitored daily throughout the experiment. At the end of week 16, rats were humanely euthanized with an overdose of sodium pentobarbital. Five milliliters of blood samples were then collected via cardiac puncture, centrifuged at 4000 rpm for 10 min. Liver tissues were carefully collected, weighed, rapidly frozen in liquid nitrogen, and stored at −80 °C for further analysis.

### 2.3. Measurement of Fasting Blood Glucose and Insulin

After a 12 h fasting period, blood samples were collected from the rats’ tail veins the following morning, and fasting blood glucose (FBG) levels were measured using a glucometer (Omron Healthcare, Lake Forest, IL, USA). Serum insulin levels were assessed using commercially available enzyme-linked immunosorbent assay (ELISA) kits (Jiangsu Jingmei Biotechnology Co., Ltd., Yancheng, China).

### 2.4. Serum Biochemical Analysis

Total triglyceride (TG), total cholesterol (TC), low-density lipoprotein cholesterol (LDL-C), and high-density lipoprotein cholesterol (HDL-C) levels were determined using an assay kit (Nanjing Jiancheng Bioengineering Institute, Nanjing, China).

### 2.5. Lipid Accumulation Analysis

Briefly, liver tissue samples were sectioned and subsequently stained with Oil Red O (Beyotime, Beijing, China) to evaluate lipid accumulation. The stained sections were then examined under an optical microscope (BX53F; Olympus, Tokyo, Japan).

### 2.6. Analysis of Antioxidant Status and Serum Adipokine Levels

Serum malondialdehyde (MDA), superoxide dismutase (SOD), and total antioxidant capacity (TAC) levels were determined using assay kits (Nanjing Jiancheng Biological Technology Institute, Nanjing, China). Serum levels of leptin (LEP) and adiponectin (ADP) were assessed using commercially available ELISA kits (Jiangsu Jingmei Biotechnology Co., Ltd., Yancheng, China).

### 2.7. RNA Extraction and Transcriptome Analysis

Total RNA was isolated from liver samples using TRIzol reagent (Invitrogen, Carlsbad, CA, USA) following the steps in the instructions. The quantity and purity of the extracted RNA were evaluated using a NanoDrop ND-1000 spectrophotometer (NanoDrop, Wilmington, DE, USA). The fragmented RNA was converted into complementary DNA (cDNA) by SuperScript II Reverse Transcriptase (Invitrogen, Carlsbad, CA, USA). The complex duplexes of DNA and RNA were converted into DNA duplexes using *E. coli* DNA polymerase I (#M0209L, New England Biolabs, Ipswich, MA, USA) and Ribonuclease H (#M0297, New England Biolabs, Ipswich, MA, USA). The synthesized duplexes were treated with a dUTP solution (Thermo Fisher, San Jose, CA, USA) to blunt the ends of the double-stranded DNA. An A-base was appended to both extremities of each fragment to facilitate ligation with a connector possessing a complementary T-base. Subsequently, the fragment size was filtered and purified utilizing magnetic beads. The second-strand cDNA underwent digestion with UDG enzyme (#M0280, New England Biolabs, Ipswich, MA, USA), initially pre-denatured at 95 °C for 3 min via PCR, followed by denaturation at 98 °C across 8 cycles of 15 s each, annealing at 60 °C for 15 s, extension at 72 °C for 30 s, culminating in a final extension at 72 °C for 5 min, thereby generating a library comprising fragments measuring approximately 300 ± 50 bp. An Illumina NovaSeq^TM^ 6000 (LC Bio Technology Co., Ltd., Hangzhou, China) was utilized for paired-end sequencing in PE150 sequencing mode, according to standard procedure.

### 2.8. Untargeted Hepatic Metabolome Analysis

Briefly, liver samples were gently thawed on ice, and metabolites were extracted using 50% methanol buffer. Additionally, pooled quality-control (QC) samples were prepared in the same volume by combining 10 μL of each extraction mixture [13]. All the samples were analyzed using an LC-MS system. A Thermo Scientific^TM^ Q Exactive^TM^ HF Hybrid-Quadrupole Orbitrap^TM^ mass spectrometer (Thermo Fisher Scientific, Waltham, MA, USA) was utilized to detect the metabolites eluted from the column. It operated in both positive and negative ion modes.

The LC-MS raw data underwent several preprocessing steps using XCMS software (version 3.7.1), which included peak selection, grouping, retention time correction, secondary peak grouping, and the annotation of isotopes and adducts. Data files were converted to mzXML format via MSConvert (ProteoWizard version 3.0) and then processed with the XCMS (version 3.7.1), CAMERA (version 1.38.1), and metaX (version 1.4.2) toolbox included in the R software 3.1.1 (Lucent Technologies, Jasmine Mountain, USA). Ions were identified by combining retention time and m/z data, and their intensities recorded in a three-dimensional matrix with peak indices, sample names, and ion intensity information. Metabolites were identified using online KEGG, HMDB, and SMPDB databases; those with a mass difference less than 10 ppm from observed values were annotated and validated through isotopic distribution measurements, further confirmed with an in-house fragment spectrum library.

To enhance data quality, the peak intensities were preprocessed using MetaX (version 1.4.2), excluding features detected below 50% of QC samples or 80% of biological samples. Missing values in remaining peaks were imputed with the k-nearest neighbor algorithm. Principal component analysis (PCA) was employed for outlier detection and batch effect evaluation. A quality control-based robust LOESS signal correction minimized signal intensity drift over time. Metabolic features with relative standard deviations exceeding 30% across QC samples were removed, ensuring reliable metabolite identification and quantification in the untargeted metabolomics analysis [14].

### 2.9. Real-Time Quantitative PCR

All samples were analyzed using reverse transcription polymerase chain reaction (RT-PCR) to validate the transcriptomic results. Briefly, the RNA samples used for transcriptome analysis were reverse transcribed with a PrimeScript™ RT Reagent Kit with gDNA Eraser (Perfect Real Time; TaKaRa, Dalian, China). RT-PCR was conducted on a StepOnePlus Real-Time PCR System (Applied Biosystems, Waltham, MA, USA) using TB Green^®^ Premix Ex Taq™ II (Tli RNaseH Plus) (TaKaRa Co., Ltd., Dalian, China). The primer sequences used in the RT-PCR are listed in Table S1.

### 2.10. Western Blot Analysis

Samples were homogenized in RIPA lysis buffer (Beyotime, Beijing, China) containing PMSF (1:100; Aladdin, Shanghai, China), and a phosphatase inhibitor cocktail (1:50; Beyotime, Beijing, China) on ice for 30 min. Following this, the samples were centrifuged at 12,000 rpm at 4 °C for 5 min. The protein content of the supernatant was then quantified using a BCA assay kit (Beyotime, Beijing, China). Equal amounts of protein samples were separated on SDS-PAGE and subsequently transferred onto PVDF membranes. After blocking with a blocking reagent, the membranes were incubated with the following primary antibodies: IRS-2 (DF7534, Affinity Biosciences, Changzhou, China), p-PI3K (17366S, Cell Signaling Technology, Danvers, MA, USA), PI3K (ab154598, Abcam, Waltham, MA, USA), p-AKT (4060S, Cell Signaling Technology, Danvers, MA, USA), and AKT (4685S, Cell Signaling Technology, Danvers, MA, USA) (1:1000 dilution) at 4 °C overnight. After washing, the membranes were incubated with appropriate HRP-conjugated secondary antibody (1:50,000 dilution) at room temperature for 2 h. After 5 additional TBST washes, the membranes were incubated with ECL substrate solution (Beyotime, Beijing, China) and visualized using an autoradiography film.

### 2.11. Statistical Analysis

The data are presented as mean ± standard deviation and analyzed with SPSS 16.0 software (SPSS Inc., Chicago, IL, USA). To assess statistical significance among different groups, one-way analysis of variance (ANOVA) was utilized, followed by Duncan’s multiple range test. A significance level of *p* < 0.05 was established, with *p* < 0.01 regarded as highly significant. For the metabolome analysis, each metabolite’s relative abundance was log-transformed prior to analysis to achieve normality. PCA of the normalized data was conducted using R software (3.1.1). Differential metabolites were identified based on the following criteria: variable importance in project (VIP) ≥ 1, fold change ≥2 or ≤0.5, and *p* < 0.05 as determined by orthogonal partial least squares–discriminant analysis (OPLS-DA).

## 3. Results

### 3.1. Effect of HB on HFD-Induced Obesity in Rats

**Figure 2:**
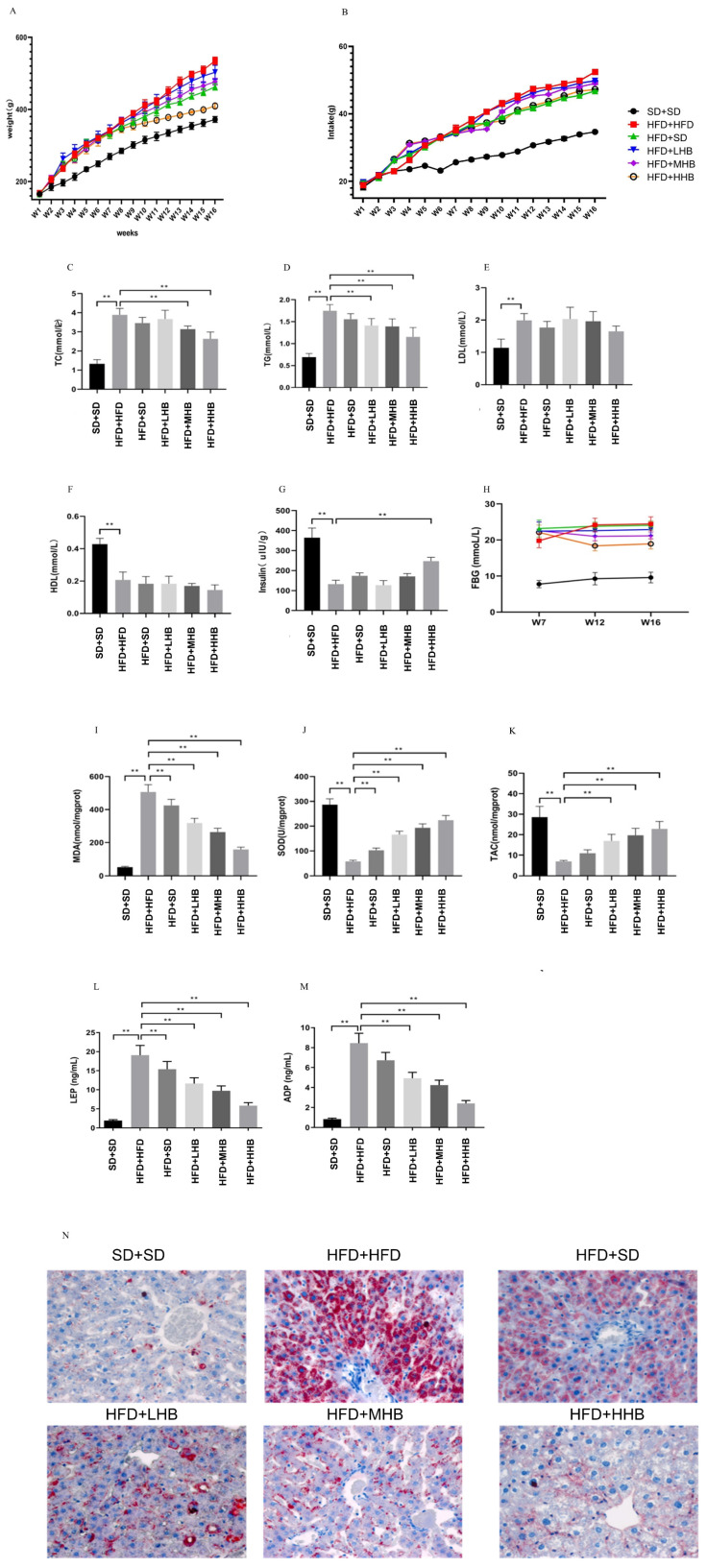
Effect of highland barley (HB) on body weight (**A**), feed intake (**B**), serum total triglyceride (TG) (**C**), total cholesterol (TC) (**D**), low-density lipoprotein cholesterol (LDL-C) (**E**), high-density lipoprotein cholesterol (HDL-C) (**F**), insulin (**G**), fasting blood glucose (FBG) (**H**), malonaldehyde (MDA) (**I**), superoxide dismutase (SOD) (**J**), total antioxidant capacity (TAC) (**K**), leptin (LEP) (**L**), adiponectin (ADP) (**M**), and liver tissues (**N**) in obese rats. Representative liver tissue samples were stained with Oil Red O. ** *p* < 0.01.

Rats in the HFD+HFD group exhibited a considerable increase (*p* < 0.05) in body weight compared to those in the SD+SD group (Figure 2A). While the HFD+SD group showed a slight reduction in body weight relative to the HFD+HFD group, the introduction of HB at all three supplementation levels markedly mitigated (*p* < 0.05) the HFD-induced weight gain across these groups, with the greatest suppression observed in the HFD+HHB group. Regarding feed intake, the HFD+HFD group consumed significantly more (*p* < 0.05) than the SD+SD group. Conversely, both the HFD+SD and HB-supplemented groups demonstrated varying degrees of reduced feed intake compared to the HFD+HFD group (Figure 2B).

Additionally, HB supplementation at 45 and 60% significantly attenuated (*p* < 0.01) the HFD-induced increase in serum TG and TC levels, but did not affect HDL and LDL levels (Figure 2C–F). HFD+STZ led to a substantial rise in fasting glucose levels and a reduction in insulin levels when compared to the SD+SD group. Conversely, HB supplementation improved glucose homeostasis by significantly increasing (*p* < 0.01) insulin levels and decreasing glucose levels, with the HFD+HHB group showing the most significant effect (Figure 2G,H).

Furthermore, HFD significantly elevated (*p* < 0.01) MDA levels and reduced both TAC and SOD (Figure 2I–K). However, HB supplementation upregulated SOD activity, improved TAC, and reduced MDA levels in rats with obesity. Additionally, HB supplementation significantly attenuated (*p* < 0.01) HFD-induced increase in serum LEP and ADP levels, with the HFD+HHB group showing the most significant effect (Figure 2L,M). Histological analysis of the liver tissue indicated an increased accumulation of red neutral fat particles in hepatocytes and large red lipid droplets in the cytoplasm in the HFD+HFD group. However, hepatic fat accumulation showed a decreasing trend in the HFD+SD and HB groups. Notably, there was a significant decrease in hepatic fat accumulation in the HFD+HHB group (Figure 2N).

### 3.2. Effect of HB on Hepatic Transcriptome of Rats

**Figure 3:**
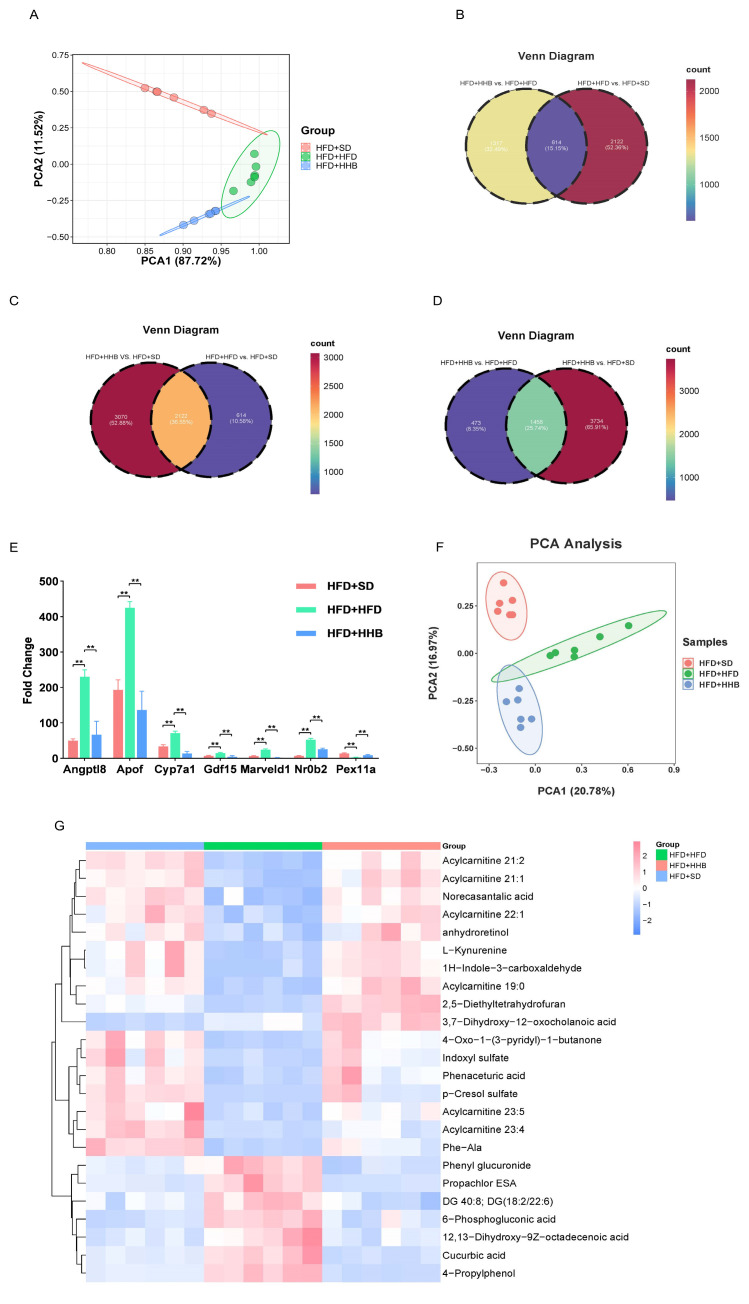
Scatter plot of PCA for differentially expressed genes (DEGs) in the hepatic tissues of rats across the HFD+SD, HFD+HFD, and HFD+HHB groups (**A**). Venn plot showing overlapping DEGs in the HFD+HHB vs. HFD+HFD and HFD+HFD vs. HFD+SD (**B**), HFD+HHB vs. HFD+SD and HFD+HFD vs. HFD+SD (**C**), HFD+HHB vs. HFD+HFD and HFD+HHB vs. HFD+SD groups (**D**). Transcriptomic analysis of DEGs (**E**). Scatter plot of PCA for metabolites in the hepatic tissues of rats across the HFD+SD, HFD+HFD, and HFD+HHB groups (**F**). Heatmap of identified metabolites in the HFD+SD, HFD+HFD, and HFD+HHB groups (**G**). ** *p* < 0.01.

RNA sequencing was performed on liver samples from the HFD+HHB, HFD+SD, and HFD+HFD groups. PCA revealed that the gene expression among the three groups was distinct (Figure 3A). Differential expression analysis identified 1931 differentially expressed genes (DEGs) between the HFD+HHB and HFD+HFD groups, as well as 2736 DEGs between the HFD+SD and HFD+HFD groups. Among these DEGs, 614 were shared between both the HFD+SD and HFD+HHB groups (Figure 3B). HFD significantly upregulated 1678 genes and downregulated 1058 genes compared to those in the HFD+SD group. Conversely, HB supplementation upregulated 1355 genes and downregulated 576 genes compared to those in the HFD+HFD group.

Based on the sequencing results, the significantly affected genes involved in liver metabolism were screened. Angptl8, Apof, cholesterol 7 alpha-hydroxylase (CYP7A1), growth differentiating factor 15 (GDF15), Marveld1, and Nr0b2 were significantly upregulated after HFD and significantly downregulated after HB supplementation at 60%. Pex11a was significantly downregulated after HFD and significantly upregulated after HB supplementation at 60% (Figure 3E).

### 3.3. Effect of HB on Hepatic Metabolic Profiles of Rats

PCA showed an apparent separation between the three groups (Figure 3F). Small molecular pathway database (SMPDB) analysis showed that differentially expressed metabolites (DEMs) among the three groups were mainly enriched in the pentose phosphate pathway, Warburg effect, and tryptophan metabolism. Additionally, heatmap visualization showed an inverse trend in the levels of DEMs between the HFD+HFD and HFD+SD groups. However, HB supplementation reversed HFD-induced changes to some extent. Overall, these results indicate that HB may reverse HFD-induced changes in the metabolic process in obesity (Figure 3G).

### 3.4. Integrated Transcriptomic and Metabolomic Analyses Indicated Candidate Genes Related to Glycolipid Metabolism

Integrated transcriptome and metabolome analysis was performed to further explore the genes involved in metabolic pathways within the livers of obese rats. In total, 57 genes were identified as being associated with metabolic pathways in the liver of rats with obesity (Supplementary Table S2). Additionally, 27 genes were common to the HFD+SD vs. HFD+HFD and HFD+HHB vs. HFD+HFD groups (Supplementary Table S3). On the basis of the findings from the integrated transcriptome and metabolome analysis, it could be speculated that HB may alleviate obesity by modulating pathways involved in glycolipid metabolism and energy homeostasis. To validate this hypothesis, the expression levels of glycolipid metabolism-related genes were examined, including ABCG8, CYP2C12, CYP2C24, CYP7A1, and IRS2. Notably, the genes showed opposite trends in their expression levels between HFD+SD and HFD+HFD groups, as well as between HFD+HHB and HFD+HFD groups.

**Figure 4:**
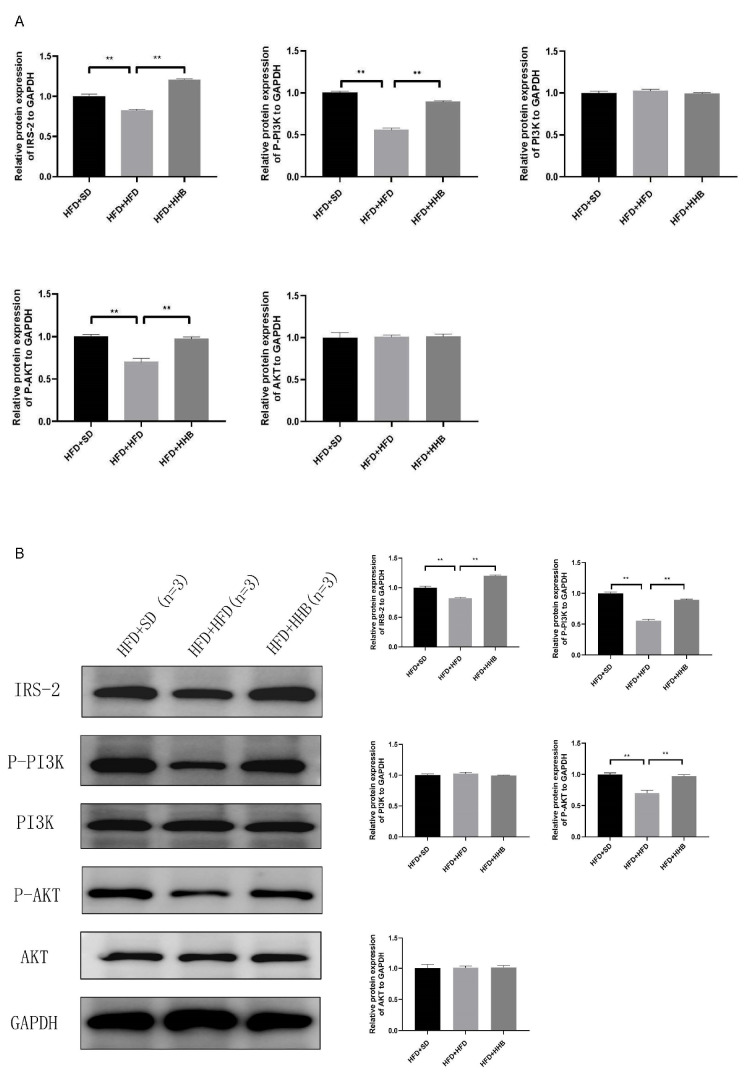
Effect of HB on relative mRNA expression of ABCG8, CYP2C12, CYP2C24, CYP7A1, and IRS2 (**A**). Effect of HB on the expression of proteins in the IRS2/PI3K/AKT signaling pathway (**B**). ** *p* < 0.01.

To elucidate the potential anti-obesity mechanism of HB, we identified significantly altered metabolic pathways in the livers of obese rats following HB supplementation. Additionally, RT-PCR was employed to validate the expression of selected genes involved in the hepatic metabolism. RT-PCR showed that ABCG8, CYP2C12, CYP2C24, CYP2C12, and IRS2 mRNA expression were significantly lower in the HFD+HFD group compared to the HFD+SD group, whereas the mRNA expression of CYP7A1 was significantly higher. Compared to the HFD+HFD group, ABCG8, CYP2C12, CYP2C24, CYP2C12, and IRS2 mRNA expression was significantly increased and CYP7A1 expression was decreased in the HFD+HHB group (Figure 4A). Moreover, RT-PCR showed that the expression trends of ABCG8, CYP2C12, CYP2C24, CYP7A1, and IRS2 in each group were consistent with the sequencing results.

### 3.5. Effect of HB on the IRS2/PI3K/AKT Signaling Pathway In Vivo

To investigate the underlying mechanism of HB’s anti-obesity effect, the expression level of proteins involved in the IRS2/PI3K/AKT signaling pathway was determined. In comparison to the HFD+SD group, the HFD+HFD group exhibited a significant downregulation of IRS2, p-PI3K, and p-AKT protein levels; however, no considerable differences were noticed in the expression of PI3K and AKT proteins between these groups. Notably, HB supplementation at 60% significantly upregulated IRS2, p-PI3K, and p-AKT protein levels, but did not affect PI3K and AKT expression (Figure 4B).

## 4. Discussion

In this study, we explored the anti-obesity properties and mechanisms of HB in rats fed an HFD. Obesity is known to elevate the risk of T2DM and its related complications. We developed a rat model of obesity by feeding an HFD for 6 weeks and administering STZ intraperitoneally on the fifth day to shorten the development time. This combined approach was used to create a stable model with abnormal glucose metabolism within a shorter period, as inducing obesity with an HFD alone is time-consuming. Rats were fed an HFD with a single administration of a low dose of STZ that could partially damage pancreatic beta cells, leading to elevated blood glucose and simulating the pathogenesis of T2DM. Our model demonstrated significant increases in food intake and body weight, along with markedly reduced insulin levels and significantly elevated fasting blood glucose levels, effectively replicating key features of obesity and T2DM. Multiple studies have suggested the favorable effects of highland barley on obesity, hyperlipidemia, and hyperglycemia [15,16,17,18]. In this study, HB supplementation effectively prevented HFD-induced insulin resistance. Notably, improvements induced by HB in insulin sensitivity, fasting blood glucose levels, leptin, and adiponectin effectively prevented insulin resistance, reduced weight gain, and improved glycolipid metabolism and antioxidant capacity in rats with obesity. Compared with the low (30%) and medium (45%) doses, the high (60%) dose of HB prevented insulin resistance. The liver serves as a primary target organ for insulin, occupying a crucial position in the maintenance of glucose homeostasis. Insulin directly acts on hepatocytes to regulate various metabolic pathways, including glycogenesis and glucose production, via glycogenolysis and gluconeogenesis [19]. Recent studies have revealed potential mechanisms through which dietary interventions like HB may modulate insulin sensitivity and improve glucose metabolism. These mechanisms encompass the attenuation of inflammation, enhancement of antioxidant defenses, and regulation of gut microbiota composition. Each of these factors plays a vital role in insulin signaling pathways, thereby contributing to the overall enhancement of metabolic health observed with HB supplementation [20,21,22].

Hepatic transcriptome analysis was conducted to elucidate the effects of HB on hepatic metabolic processes. Transcriptome analysis showed that a high-dose HB intake significantly counteracted the HFD-induced increase in the expression of six hepatic metabolism genes (Angptl8, Apof, CYP7A1, GDF15, Marveld1, and Nr0b2) and upregulated Pex11a expression. Angptl8 regulates lipid metabolism and participates in diseases, such as renal insufficiency and metabolic-associated fatty liver disease [23,24,25]. Apof plays a role in regulating LDL cholesterol levels, particularly under conditions where LDL is either insufficient or not functioning efficiently [26]. Additionally, CYP7A1 catalyzes the generation of HDL [27,28,29]. Moreover, both intracellular GDF15 and cyclically mature GDF15 are involved in biological processes, such as energy homeostasis and weight regulation [30,31]. Marveld1 interacts with catalase to enhance its activity and maintain its stability [32]. Furthermore, Nr0b2 participates in various metabolic processes, including drug metabolism, bile acid secretion, and glucose homeostasis [33,34]. Importantly, Pex11a knockdown impairs physical activity and energy expenditure, leading to dyslipidemia and obesity [35,36].

To investigate the potential mechanism by which HB affects obesity induced by HFD, changes in the liver transcriptome of rats in HFD+HHB, HFD+SD, and HFD+HFD groups were examined using integrated metabolome and transcriptome analysis. The HB suppressed the glycolipid metabolism by upregulating ABCG8, CYP2C12, CYP2C24, and IRS2, and downregulating CYP7A1 expression, which was verified using RT-PCR. IRS2 is involved in most metabolic processes mediated by insulin receptors. IRS2 activation promotes the phosphorylation and activation of PI3K, resulting in AKT phosphorylation and activation. Activated AKT transmits biological signals downstream of various receptor substrates, thereby inducing biological effects [37].

To elucidate the underlying mechanism of HB in insulin resistance among obese rats, we examined the protein expression of IRS2/PI3K/AKT. Western blotting showed that HB intake effectively enhanced insulin sensitivity in obese rats by increasing the IRS2, p-PI3K, and p-AKT protein levels. A HFD causes obesity in rats and increases glucose and insulin tolerance, leading to insulin resistance. An obesity-associated increase in serum TC and TG levels aggravates liver injury, increases gluconeogenesis, accelerates glycogen breakdown, and blocks the IRS2/PI3K/AKT signaling pathway. However, HB intake promoted PI3K/AKT phosphorylation, and PI3K phosphorylation transduces signals downstream of AKT. Additionally, AKT activation regulates glucose metabolism through multiple pathways. Therefore, it could be speculated that HB enhances insulin sensitivity by regulating the IRS2/PI3K/AKT signaling pathway.

Despite the promising findings, this study had some limitations. Firstly, it was conducted exclusively on male rat models, which introduces a potential sex bias and limits the direct extrapolation of the results to humans. Although rat models share many physiological and metabolic similarities with humans, significant differences may still exist in the responses to dietary functional components and metabolic pathways between males and females. Therefore, more research in clinical settings is needed in the future to validate the applicability of these findings in humans, as there may be variations in response due to factors such as lifestyle, genetics, and environmental influences. Secondly, the study primarily relied on transcriptome and untargeted metabolome analyses to explore the effects of HB on HFD-induced gene expression and metabolic changes. While these techniques provide valuable insights, they have their limitations. For instance, transcriptome analysis reveals changes in gene expression but not necessarily protein abundance or activity. Similarly, untargeted metabolome analysis, while detecting a broad range of metabolite changes, may lack depth in specific metabolic pathways. Future studies could benefit from incorporating additional technologies such as proteomics and targeted metabolome analysis for a more comprehensive understanding of the mechanisms involved. Thirdly, the study focused on the selected IRS2/PI3K/AKT signaling pathway, without exploring all potentially relevant pathways comprehensively. Obesity and its associated complications arise from complex interactions between multiple factors and networks. A single-pathway approach may not fully capture the therapeutic effects of HB. Further research should adopt a systems biology approach, considering various signaling pathways and molecular interactions for a holistic understanding of HB’s therapeutic impact. Fourthly, the dose selection and intervention duration in this study might restrict the interpretation of the results. Even though different doses of HB supplementation were tested, the optimal dosage and intervention period require further optimization. Additionally, the long-term effects remain undetermined, and future studies should consider extended follow-ups to assess the sustained impact of HB supplementation on obesity and related metabolic disturbances. Moreover, we used raw HB instead of the typically processed and heated barley consumed by humans; and the effects of HB on glycolipid metabolism may vary across different varieties. For example, Kunlun 14 was used in this study, whereas Zangqing 320 and 2000 were used in previous studies [16,17,18]. Similar to other whole grains, such as oats, HB contains several active ingredients, including polyphenols, dietary fiber, and β-glucan, which are involved in glycolipid metabolism [38,39,40]. Therefore, further research is necessary to assess the anti-obesity effects of other varieties of HB and identify the specific components involved in glycolipid metabolism regulation. It is also crucial to devise methods to enhance and preserve the quality of HB and its active ingredients.

## 5. Conclusions

Conclusively, HB alleviates HFD-induced obesity and liver injury in a rat model of obesity possibly via the IRS2/PI3K/Akt signaling pathway. Overall, these findings collectively suggest that HB holds promise as a functional diet for the prevention of abnormal glycolipid metabolism. However, further studies, particularly human clinical trials, are essential to confirm the applicability of these results and to elucidate the underlying molecular mechanisms more comprehensively.

## Figures and Tables

**Table 1:** Composition of experimental diets.

	SD (g)	HFD (g)	LHB (g)	MHB (g)	HHB (g)
Highland barley	0.0	0.0	300.0	450.0	600.0
Corn starch	397.0	0.0	200.0	100.0	0.0
Dextrin	132.0	163.5	100.0	50.0	0.0
Sucrose	100.0	89.4	100.0	100.0	100.0
Cellulose	50.0	65	0.0	0.0	0.0
Casein	200.0	260	170.0	160.0	150.0
Cystine	3.0	3.9	3.0	3.0	3.0
Soybean oil	70.0	32.5	80.0	90.0	100.0
Lard	0.0	318.5	0.0	0.0	0.0
Mineral mixture	35.0	13.0	35.0	35.0	35.0
Vitamin mixture	10.0	13.0	10.0	10.0	10.0
Choline bitartrate	2.5	2.5	2.5	2.5	2.5
Total	999.5	961.3	1000.5	1000.5	1000.5

SD, standard diet; HFD, high-fat diet; LHB, low highland barley diet; MHB, medium highland barley diet; HHB, high highland barley diet.

## Data Availability

The dataset supporting the findings of this article will be made available upon reasonable request to the corresponding authors.

## Supplementary Materials

The following supporting information can be downloaded at: https://www.mdpi.com/article/10.3390/nu16203518/s1, Table S1: RT-PCR primer sequences; Tables S2 and S3: Integrated analysis results.
