# Supplementary material for: Highland Barley Alleviates High-Fat Diet-Induced Obesity and Liver Injury Through the IRS2/PI3K/AKT Signaling Pathway in Rats
Source: Nutrients. 2024 Oct 17;16(20):3518. doi: 10.3390/nu16203518 (PMC11510035; doi:10.3390/nu16203518)
Supplement: Supplementary file 1 [file nutrients-16-03518-s001.zip › Table S1 RT-PCR primer sequences.pdf]

Table S1. RT-PCR primer sequences

Gene	Forward	Reverse
ABCG8	CAGACAAGCCGCTCTCCTTCAT	CGACCGCTCCGAGTGACATT
CYP2C12	GAACATCTGGCAATCCTGGTGACTAA	TGTGTGGGTACTTCATCAAGAGCAAA
CYP2C24	TGAGATATGGACTCTTGCTCCTACTG	GCCTCCTGTGTCTGCCAATCA
CYP7A1	CAGGTCTCTGAACTGATCCGTCTAC	AGAATAGCGAGGTGCGTCTTGG
IRS2	CCTTCTCCTCTACCACCACTGTCA	GGATGCTGTTGCCTTCACTGCTT
GADPH	AGGTTGTCTCCTGTGACTTCAA	CTGTTGCTGTAGCCATATTCATTG
